# Persistence of the Strictly Endemic Plants of Forest Margins: The Case of *Cirsium alpis-lunae* in the Northern Apennines (Italy)

**DOI:** 10.3390/plants11050653

**Published:** 2022-02-28

**Authors:** Giuseppe Fenu, Lorenzo Lazzaro, Lorenzo Lastrucci, Daniele Viciani

**Affiliations:** 1 Department of Life and Environmental Sciences, University of Cagliari, Viale S. Ignazio da Laconi 13, 09123 Cagliari, Italy; 2 Department of Biology, University of Florence, Via G. La Pira 4, 50121 Florence, Italy; lorenzo.lazzaro@unifi.it (L.L.); daniele.viciani@unifi.it (D.V.); 3 Natural History Museum, Section of Botany, University of Florence, Via G. La Pira 4, 50121 Florence, Italy; lorenzo.lastrucci@unifi.it

**Keywords:** *Cirsium*, edge specialist plant, mountain plant, narrow endemic plant, neglected plant, plant conservation

## Abstract

Narrow endemic plants constitute a pivotal group for conservation, being often reduced to a small contingent of individuals and frequently threatened. However, effective conservation actions require reliable basic information about distribution range, ecological requirements, and population traits. Nevertheless, such knowledge results are incomplete or even completely missing for some neglected or recently described plants, such as *Cirsium alpis-lunae*, a thistle exclusive to the N-Apennines (Italy). To fill this gap, all sites where *C. alpis-lunae* grow were monitored, and data on the site and population traits were collected. Our results indicated that this plant is restricted to 16 scattered sites, varied in surface area and number of individuals. Reproductive and juvenile plants showed to be affected by roughly the same variables, in particular the surface of the site, the slope aspect, and the canopy cover. The narrow ecological niche of *C. alpis-lunae* was mainly determined by the canopy cover, and where coverage increases, the number of individuals decreases. The individuals only grow at forest edges, where the peculiar ecological conditions are limiting factors for the development of forestry cover; some other factors (i.e., high inclination and instability of the substrate) contribute to limiting the development of forestry vegetation and guarantee the persistence of these ecotones. Despite the great difficulties in accessing the sites where this species grows, this study presents, for the first time, a complete picture of the *C. alpis-lunae* population and yielded important data to identify effective conservation measures.

## 1. Introduction

Population dynamics is an area of science that attempts to provide an explanation for variations over time in the observed size and structure of biological populations [1,2,3,4]; determining whether a population is growing or declining is a central issue in conservation biology [5,6,7]. In a way, all plants face the risk of extinction due to various causes, such as habitat destruction, habitat fragmentation, displacement by or hybridisation with invasive alien species, climate change, and overharvesting for economic purposes [6,8,9,10]. Plants with extremely restricted distribution ranges are particularly sensitive to these threats because of their narrow distribution and, often, low numbers of populations or individuals. On the other hand, some plant species, generally narrow endemics, by their nature have very small or scattered populations, often extremely adapted to their environment. Since narrow endemic plants are often reduced to a small contingent of individuals or are frequently threatened, they constitute a pivotal group for conservation [6,11,12]. Regardless, to be effective, conservation actions for those species require reliable basic information about the distribution ranges, ecological requirements, population numbers, population sizes, and, if possible, demographic patterns (i.e., vital rates) over a defined period of time [3,13,14,15]. Although demographic analyses have been successfully applied in plant ecology and evolutionary biology [16,17,18,19], their use in conservation biology remains lacking [15,20,21,22,23,24]; this is especially true in the Mediterranean Basin, where in-depth demographic studies on endemic and threatened plant populations are uncommon [7,15,20,25,26,27,28]. This lack of knowledge is even more relevant in the case of plants that have been neglected, recently described, or have typical or exclusive dynamic or peculiar habitats, such as ecotones.

Endemic plants restricted to ecotonal transition zones are a challenging conservation objective, and their study is highly demanding. Indeed, the switch between different habitats generates transition zones with peculiar ecological properties, which are recognised as ecotones [29,30]. Edge effects can have serious impacts on species diversity and composition, community dynamics, ecosystem functioning, and interactions [31,32,33,34]. In fact, in these transition areas, there are pronounced complex ecological gradients, which are characterised as both abiotic (e.g., where large differences in incoming radiation, wind speed and direction, temperature and humidity occur at short spatial scales) and biotic factors (composition and structure of biotic assemblages such as patterns of insect presence, abundance, and behaviour; [35,36]). Such local gradients in microclimatic conditions are especially pronounced near forest edges. These transition zones between forests and adjacent open land are characterised by a strong inflow of warm or cool air, depending on the season and the time of day [37,38]. In particular, due to abrupt changes in vegetation structure and composition [34,37], forest edge zones are characterised by environmental gradients that can extend up to 100 m into the forest interior [37,38]. For instance, during the summer, temperature and light levels decrease, whereas the relative air humidity increases from the edge towards the forest interior [38,39,40]. This makes transition zones more susceptible to temperature extremes and drought stress in comparison with forest interiors. Altered microclimatic conditions in forest edges, from warm and dry at the edge to cool and moist in the interior, create gradients in understorey biodiversity and consequently induce the establishment of typical habitats for edge-loving vegetation and organisms [41,42,43]. Edge effects are posited as important mechanisms for driving the persistence, reproduction pattern, and recruitment rate of locally adapted plants, and for this reason, studying ecological responses at habitat edges is a key topic in plant ecology and conservation [34,44,45].

*Cirsium* Mill. (Asteraceae Bercht. & J. Presl.: Cardueae Cass.) is a large genus comprising more than 450 accepted species (as many as 491 according to [46]), usually biennial or perennial spiny herbs, primarily distributed in the northern hemisphere but naturalised worldwide [14,47,48,49]. Among these, several species are widely common, but others are extremely taxonomically debated, only provisionally accepted, or simply neglected. As a general consequence, the number of deep species studies into this genus has fallen [50,51,52,53], but in other plants, even the basic knowledge is incomplete or even completely missing. An iconic plant of the latter group is *Cirsium alpis-lunae* Brilli-Catt. *et* Gubellini; it is a strictly endemic yellow-flowered thistle, growing exclusively in a small area of the northern Apennines, and was recently discovered and described [54]. Its occurrence was reported in local and national floristic and vegetation studies [55,56,57,58,59,60,61,62,63] and, only recently, the communities where it grows were investigated from synecological and syntaxonomic points of view [64]. Despite its conservation relevance highlighted by several authors [6,62], no basic study on its population size, structure, and dynamics, as well as its ecological requirements, has ever been carried out, mainly because of the extreme difficulty in accessing its typical sites of occurrence. To fill this gap, we investigated some populational traits concerning *C. alpis-lunae* in all the sites where this species has been reported, with the aims to (i) estimate the species’ distribution, structure, and population size; (ii) characterise its habitat; (iii) test the relationship among the abundances of mature and juvenile individuals of *C. alpis-lunae* and verify whether juvenile and mature individuals of *C. alpis-lunae* differ in environment requirements; and (iv) explore how the local plant community drives the abundance of this species.

## 2. Results

### 2.1. Population Size and Structure

**Figure 1:**
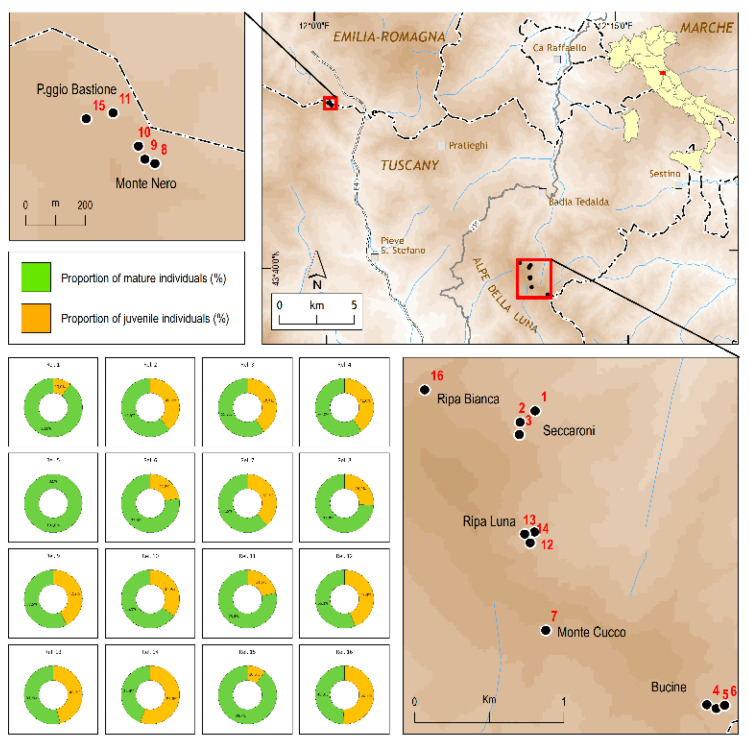
Distribution and size of the 16 localities where *Cirsium alpis-lunae* was found. The numbers reported on the map (red font) and the graphs (at the bottom left) indicate the different localities where *Cirsium alpis-lunae* grows and coincide with the numbers indicated in Table 1 (see this table for ecological and populational details of each locality).

We confirmed the presence of *C. alpis-lunae* in 16 sites (Figure 1, Table 1). The surface area of the localities varied from 20 to 1800 m^2^, at altitudes of 1118–1270 m, on slopes of 40–100%, mainly with north or northeast aspects (Table 1). Our results confirm that this plant grows on humid and unstable steep slopes, on open, well-drained earthy screes derived from sandstone–marly flysch substrata, and, according to the European Habitat Directive (DIR 92/43/EEC), participates in plant communities attributable to the habitat’s “Tall herb fringe communities of open, humid and unstable steep slopes, on earthy screes (code 6430)” (Table 1).

The size of the global population of *C. alpis-lunae* was estimated at 1114 individuals, 430 of which were reproductive plants (<40% of the total; Table 1). The consistency of each site was variable, ranging from 5 to 187 plants and from 0 to 84 reproductive individuals (Table 1); of the total, the number of mature plants in 11 localities (69% of the total) was less than 20 individuals and in five localities (31%) less than 10 (Table 1). The percentage of mature plants exceeded 50% of the total in only two sites, while in five localities, it was lower than 25%, and in one site, there were no mature individuals (Table 1). In four localities, coinciding with those with a low number of mature individuals, no recruitment was observed (Table 1).

### 2.2. Relathionships betwenn C. alpis-lunae and Environmental Parameters

**Figure 2:**
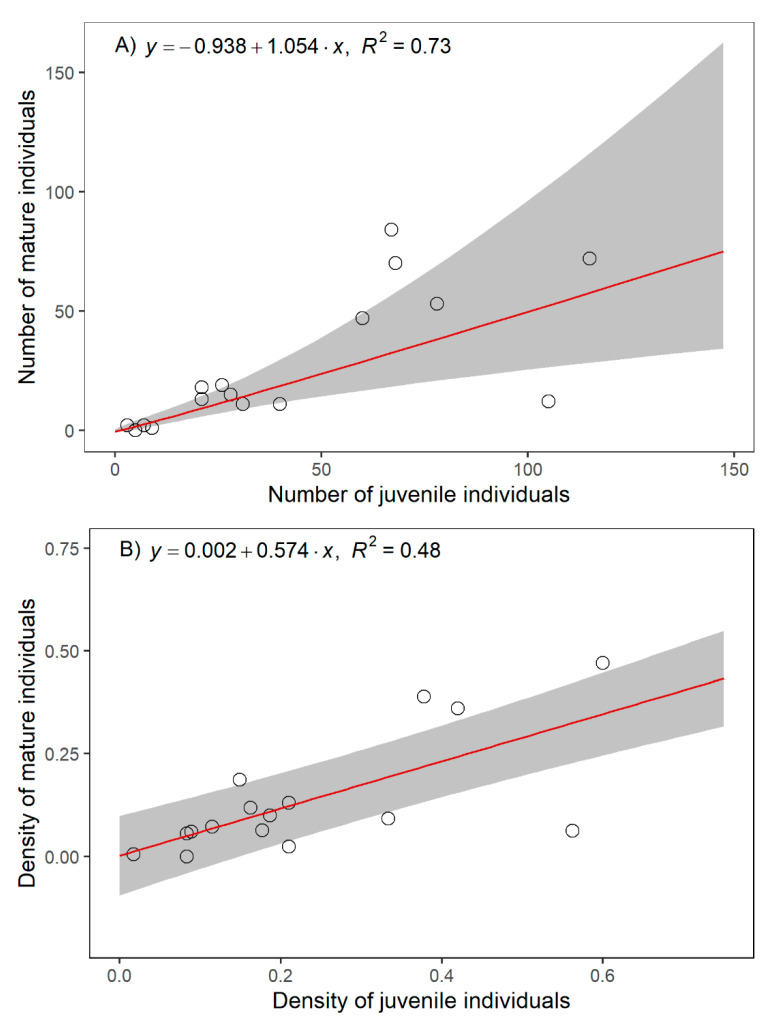
Relationship among mature and juvenile individuals of *Cirsium alpis-lunae*. (**A**) Relationship among a number of juvenile and mature individuals. Regression line parametrised in log–log spaces and back-transformed to linear scale to plot the actual numbers of the populations. (**B**) Relationship among the density of juvenile and mature individuals. Circles represent actual number and density values (in A and B, respectively) of *Cirsium alpis-lunae* mature and juvenile individuals.

The abundance of mature individuals of *C. alpis-lunae* appeared positively correlated with the abundance of juvenile ones (Figure 2A, *p* < 0.001; *F value* = 31.3, Degrees of freedom= 1, 14), with a high proportion of variance explained by the regression model (R^2^ = 0.73). The analyses of residuals and diagnostic plots for modelling the relationship resulted in the choice to apply a log transformation to both abundances. In addition, the density of the mature individuals of *C. alpis-lunae* appeared positively correlated with the density of the juvenile ones (Figure 2B, *p* < 0.001; *F value* = 44.5, Degrees of freedom= 1, 14), with a lower proportion of variance explained by the regression model compared with the model on abundance, but still valuable (R^2^ = 0.48).

**Figure 3:**
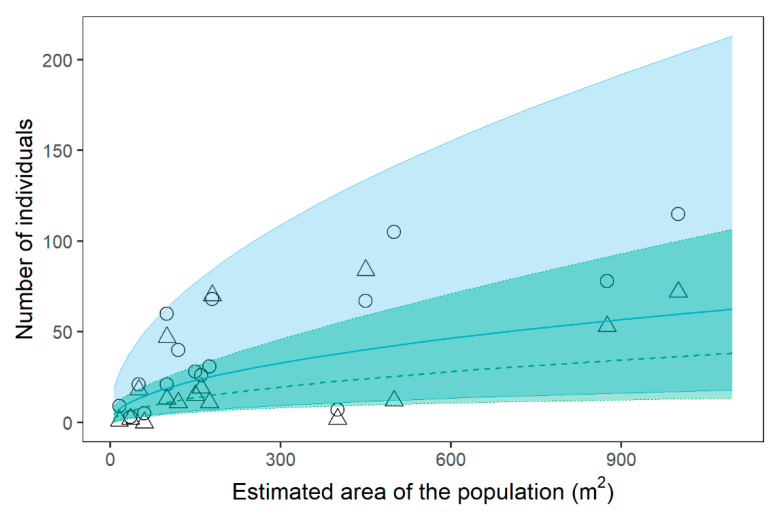
Relationship among number mature and juvenile individuals of *Cirsium alpis-lunae* and the estimated area occupied by the population. Regression lines and relative confidence intervals are calculated with the averaged coefficient following the multimodel inference in log–log spaces and back-transformed to linear scale to plot the actual numbers of the populations. Circles and solid lines = juvenile individuals; Tringles and dashed lines = mature individuals. Concerning the effect of the cover of the different vegetation layers on the cover of *C. alpis-lunae* at the subplot level, while all three factors were included in the best models, only canopy cover showed significant importance, thus resulting in the only factor actually affecting *C. alpis-lunae* (Table 2, see Table S5 for model selection table). Indeed, canopy cover negatively affected the cover of *C. alpis-lunae* at the subplot level (Figure 4).

Matures and juveniles were affected by roughly the same variables (Table 2, see Tables S1–S4 for model selection tables). In particular, *C. alpis-lunae* abundance in both stages appeared strongly related to the surface of the site, with a slight influence of slope aspect, expressed as both northness and eastness for juvenile individuals and only eastness for mature ones. Nonetheless, it should be noted that the slope aspect has low importance and low weight in multimodel averaging, resulting in a very small coefficient, highlighting the fact that its effect is negligible. As to the surface area of the site, matures and juvenile individuals showed similar coefficients, thus displaying a comparable positive dependence on the size of the site (Figure 3). As to the density of matures and juveniles, again, the slope aspect is somehow included in the models (Table 2), with both northness and eastness included in the best models for juvenile individuals, but only northness for mature ones. Again, both factors showed quite a negligible relative importance.

## 3. Discussion

Biodiversity is declining globally [9,65], and current estimates indicate that 39% of plant species are at risk of extinction [10]. However, despite agreed national and international conservation efforts, there is no evidence that the global loss of biodiversity is decelerating [65,66]. This alarming situation significantly affects many endemic plant species worldwide, and the protection of such target species and their habitats is one of the most urgent tasks in biodiversity conservation, especially in a global hotspot such as the Mediterranean Basin [67,68]. To plan effective conservation measures when needed for plants of conservation interest, basic information about the distribution ranges, ecological requirements, population numbers, population sizes, and, if possible, demographic patterns (i.e., vital rates) is required [14,15,21,69]. However, omitting the complex studies of population dynamics, plant population monitoring programmes are also particularly scarce because they are time- and resource-consuming and are therefore restricted to a few threatened species [11,23,24,70]. Thus, the knowledge about the biology, ecology, and conservation status of most plant species remains remarkably poor; this lack of knowledge is even more relevant in the case of plants neglected, recently described, typical or exclusive of dynamic or peculiar habitats, such as ecotones, or in the case of plants restricted to small and scattered populations, growing on microhabitats difficult to access, such as rocky sites [14,23,24,68].

Our study on *C. alpis-lunae* represents the first attempt to fill this gap for a narrow endemic plant restricted to a small peculiar ecological context. The results of our field investigation showed that *C. alpis-lunae* is restricted to two macroareas, as previously reported [62], in which there are 16 scattered localities where this plant always occurred in the same ecological conditions. The *C. alpis-lunae* populations mainly consist of a low number of plants, generally restricted to small surface areas at the edge of wooded areas. Despite the great difficulties in accessing the sites where this species grows, this study presents, for the first time, a complete and exhaustive picture of the *C. alpis-lunae* populations, although a margin of uncertainty in the numbers must be considered due to the difficulties in accessing the investigated sites [64].

The few existing studies on the population dynamics of chasmophytes suggest unusually stable population sizes and high local population persistence, mainly due to the conservative nature of such habitats [21,69,71,72], but this does not seem to be a general rule [23,24]. Small populations can exhibit strong annual fluctuations in size due to stochastic forces (e.g., environmental stochasticity), and environmental changes constrain the survival of single plants and start the progress of consecutive decline of individuals within the population of chasmophytic plants [23,24]. In our study case, the stability of the populations seems to guarantee the local persistence of the populations themselves, although the possibility of stochastic events occurring was not quantifiable.

The population structure (in terms of abundance) appeared strongly related to the surface area of each site. The number of adult individuals was generally low, with a few exceptions being under the minimum threshold of 20 plants in 11 populations, considered the quasi-extinction threshold generally adopted in demographic studies to help minimise the demographic stochasticity associated with a small population size [3,22]. This situation makes some populations prone to local extirpation in the near future, as well as for a single environmental stochastic event [23,24,69,73]. This finding was also supported and enhanced in four populations by the absence of recruitment; all of our data indicated a probable local extirpation of this species in a short time period. This general pattern is typical of numerous endemic plant species in the Mediterranean mountains [23,24], characterised by small populations that persist in very restricted ecological contexts. In *C. alpis-lunae*, this ecological niche is mainly determined by the canopy cover; there was a significant negative correlation between canopy cover and cover of *C. alpis-lunae*, so that where coverage increased, the number of individuals decreased and, as a direct consequence, the greater the edge surface, the greater the extension of the ecological niche and therefore the population size.

The open areas colonised by *C. alpis-lunae* are generally in contact with neighbouring Fagus sylvatica-dominated woods [64]. In this context, the *C. alpis-lunae* populations were at a small-scale dynamic equilibrium due to the ecological heterogeneity found at forest edges and mediated between the small population size and the peculiar ecological conditions that limit the development of canopy cover. Some factors seemed to contribute to keeping this situation stable, particularly the high inclination and instability of the substrate, which have become limiting factors for the development of forestry vegetation and guaranteeing the existence of these ecotones. Shifts in the relative importance of those factors could negatively influence the persistence of this plant across such an ecological gradient. Such patterns reveal that, in edge habitats, plants can rely on a very small ecological space to optimise their persistence. Thus, *C. alpis-lunae* could be considered a forest edge specialist, acting as an edge opportunist and colonising new cool and semishady niches where it can persist for a long time, as reported for other extremely specialised species particularly adapted to microecological situations [74].

However, two additional aspects should be considered in this case: the limitations in the reproductive process and the effect of ongoing climate change in mountain environments. In the first case, it would be a question of evaluating how many from the low number of mature individuals associated with the small and scattered populations act on the potential persistence of the species in the wild (in terms of genetic variability/impoverishment). On the other hand, the populations of *C. alpis-lunae* are generally composed of a small number of individuals adapted to the “cold” mountain scree slopes, making this plant species particularly fragile to warmer conditions, especially due to its stenoicity and limited dispersal capabilities. Generally, the ability of plant populations to survive at forest edges has been related to their autecology in an undisturbed or equilibrium context and the quality of ecological niches derived from sunny–shady gradients [23,24,75,76]. The habitat conditions that constitute the refugia of many cold-adapted mountain species are expected to experience a fast reduction in high mountains with increasing temperatures [77], and such habitat reduction can severely impact effective population sizes with further deleterious genetic consequences on populations. In fact, an altered microclimatic environment around forest edges could directly influence plant reproduction and recruitment [78]. Accordingly, the microclimatic conditions of the mountain scree slopes where *C. alpis-lunae* grows could have historically played, and probably still play, a key role in the long-term persistence of many species. Thus, these habitats should be protected with special measures to preserve the postglacial relict flora that inhabit these peculiar landforms. Currently, the two areas where this plant grows are included in Special Areas of Conservation (Natura 2000 codes: IT5180010, IT5180006, IT5310010), but this measure per se will probably not be sufficient to guarantee the persistence of the species in the global warming scenario, and practical actions will have to be planned and implemented. In this case, conservation measures should be planned on a small spatial scale since each population could need different conservation measures depending on the peculiar local conditions [7,79].

## 4. Materials and Methods

### 4.1. Study Species

*Cirsium alpis-lunae* is a perennial megaforbic plant, morphologically and taxonomically similar to other European and Italian *Cirsium* species such as *C. erisithales*, *C. carniolicum*, *C. oleraceum*, *C. spinosisimum*, and *C. bertolonii*. It is a scapose hemicryptophyte that grows to a height of 35 (100) cm. The flowering period is from March to July, often in August at higher altitudes or in colder aspects, and the fruiting season runs from July to August [62]. *Cirsium alpis-lunae* typically lives on humid and unstable steep slopes, on open, well-drained earthy screes derived from sandstone–marly flysch substrata, at altitudes between 1100 and 1300 m a.s.l. *C. alpis-lunae* needs a good water availability, mainly supplied by the rainfalls, and, according to its ecological requirements, it can be considered markedly meso-hygrophilous, microthermal, and rather nitrophilous [54,64]. The open areas colonised by *C. alpis-lunae* are generally small and in contact with neighbouring forests dominated by *Fagus sylvatica* [54,64].

*Cirsium alpis-lunae* is only present in two small areas of the northern Apennines, in the “Alpe della Luna” massif and in the “Monte Nero” massif, located near the border between Tuscany, Emilia-Romagna, and Marche administrative regions (Figure 1); all the known sites are geographically located near the Apennines ridge, but always on the Adriatic side of the Italian peninsula. The study area lies in a Temperate Oceanic Bioclimate [80]. Geological substrates are mainly constituted by sandstones, siltstones, and marls, generally rich in Ca and nutrients [81,82].

### 4.2. Data Collection and Preparation

We visited all sites where *C. alpis-lunae* has been reported to grow. The locations of the surveys are shown in Figure 1. All the sites were surveyed in July to minimise seasonal differences (main information on the sampling sites are reported in Table 1). For each site, we collected information regarding the site characteristics: slope, slope aspect, altitude, and substratum. To allow its inclusion in the following modelling, the slope aspect was transformed into northness and eastness according to the formulas: northness = cosine ((aspect in degrees* π)/180)) and eastness = sine ((aspect in degrees* π)/180))). Hence, at each site, we collected information regarding the *C. alpis-lunae* population, including the surface area of the total population, the number of mature (flowering) individuals of *C. alpis-lunae*, and the number of juvenile (not flowering) individuals. Finally, at each site, we performed a vegetation survey on a surface area of 16 m^2^, considered suitable to represent the local plant community, collecting the total percentage cover of the ground layer (hereafter ground cover), the total percentage cover of the intermediate layer (shrubs and young trees, hereafter shrub cover), and the total percentage cover of the upper layer (canopy level, hereafter canopy cover).

### 4.3. Data Analyses

To assess the relationship between the abundances of mature and juvenile individuals of *C. alpis-lunae,* we fitted a Generalised Least Squares model (GLSm), accounting for a Gaussian spatial correlation of the observations, using the abundance of mature *C. alpis-lunae* individuals as the response variable and the abundance of juvenile ones as the predictor. Both abundances of mature and juvenile individuals were log-transformed to obtain the normality of residuals. To disregard the possible effect of population size, we fitted a second GLSm with a consistent structure for the spatial autocorrelation but evaluated the relationship among the density of mature and juvenile individuals (i.e., number of individuals/estimated area occupied by the population).

We verified the differential requirements of juvenile and mature individuals of *C. alpis-lunae* by fitting and comparing two separate series of models describing the relationship between the *C. alpis-lunae* and the site characteristics, again using GLSm, accounting for the spatial autocorrelation of the observations. We used, as explanatory variables, all the variables measured in the field at the site level (i.e., altitude, slope, slope aspect expressed as northness and eastness, substrate, and estimated area of the population (see Table 1)). We fitted separate models per mature and juvenile population, studying, as response variables, again, both the abundance of mature and juvenile individuals and their density (as described above). In the latter case, the estimated area of the population was not included as an explanatory variable, as it is directly used in the calculation of population densities. To avoid model overfitting in such a small dataset, we used the framework of multimodel inference through the Information-Theoretic Approach [83] to select a set of “best models” and allow the evaluation of only a few predictors from all those taken into account in the study. This approach allowed the selection of the best combination of predictors from the global model, including all possible combinations. The model comparisons were performed by adopting the corrected Akaike Information Criterion (AICc), and the model choice was made based on ΔAICc (which represents the difference between each model and the most parsimonious model; see supplementary materials for details). We selected all the models with a ΔAICc with values < 4, considered to be equally parsimonious [83]. According to this procedure, only a small subset of predictors was selected as significantly affecting the response variables, and the correlation coefficients of each predictor were averaged among the selected best-fitting models. The significance of the estimated coefficient was calculated with a *z* Wald test. We estimated the relative importance w*_+(j)_* of each predictor *j* as the sum of the AICc weights across all models in which the selected predictor appeared [83]. Predictors with higher w*_+(j)_* (i.e., closer to one) have a higher weight of evidence to explain the response variable with the given data (i.e., higher relative importance). Models for the comparison procedure were fitted with maximum likelihood (ML) for the estimation of variance components, while selected models were refitted with restricted maximum likelihood (REML) for the estimate of coefficients.

To explore how the local plant community drives the abundance of this species, we also evaluated whether the cover of *C. alpis-lunae* (at the subplot level) was affected by the cover of the different vegetation layers (i.e., ground, shrub, and canopy cover). Again, we used the previous approach of multimodel inference through the Information-Theoretic Approach [83] on a series of Generalized Least Squares models accounting for a Gaussian spatial correlation of the observations, following the methods described above.

All analyses were performed in R vers. 3.4.3 [84]. The multimodel comparisons and inference were performed using the *MuMIn* package [85]. The graphs were drawn using the *ggplot2* package [86].

## Figures and Tables

**Figure 4:**
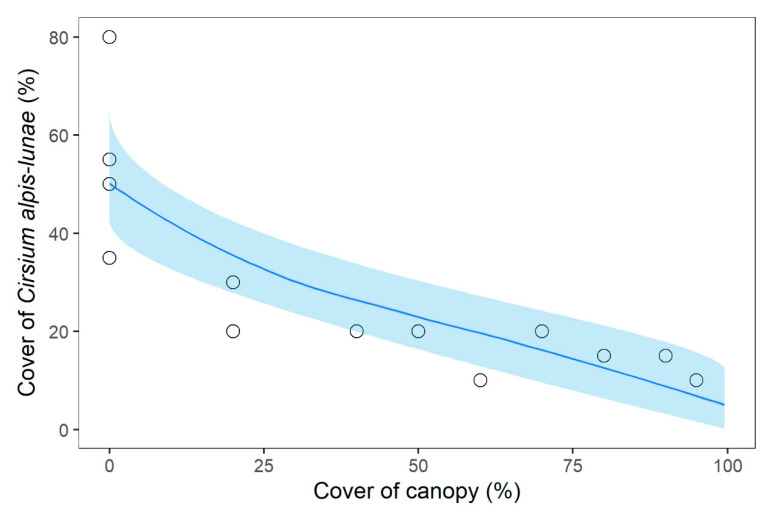
Relationship between the cover of *Cirsium alpis-lunae* and the cover of the canopy layer. Regression lines and relative confidence interval are calculated with the averaged coefficient following the multimodel inference after arc-sine transformation for response and explanatory variables and back-transformed to plot the cover percentage scale. Circles represent the actual cover values of *Cirsium alpis-lunae*.

**Table 1:** Main features of sampling sites of *Cirsium alpis-lunae* populations recorded on the field and used in the analyses.

No.	Locality (Municipality Administrative Province)	Altitude (m a.s.l.)	Slope (%)	Aspect	Estimated Area (m^2^)	Total n. of Plants	N. of Mature Plants	Recruitment
1	Seccaroni 1 (Alpe della Luna), Badia Tedalda, Arezzo	1118	100	N-NE	20	10	1	absent
2	Seccaroni 2 (Alpe della Luna), Badia Tedalda, Arezzo	1132	>100	N	1000	187	72	present
3	Seccaroni 3 (Alpe della Luna), Badia Tedalda, Arezzo	1180	>100	N	875	131	53	present
4	Bucine 1 (Alpe della Luna), Badia Tedalda, Arezzo	1190	100	NE	36	5	2	absent
5	Bucine 2 (Alpe della Luna), Badia Tedalda, Arezzo	1170	100	NE	60	5	0	absent
6	Bucine 3 (Alpe della Luna), Badia Tedalda, Arezzo	1120	100	NE	40	9	2	absent
7	Cucco (Alpe della Luna), Sansepolcro, Arezzo	1270	100	SE	100	34	13	present
8	Monte Nero 1 (Monte Nero-Poggio Bastione), Verghereto, Forlì-Cesena	1149	75	NE	175	42	11	present
9	Monte Nero 2 (Monte Nero-Poggio Bastione), Verghereto, Forlì-Cesena	1154	>100	NE	160	45	19	present
10	Monte Nero 3 (Monte Nero-Poggio Bastione), Verghereto, Forlì-Cesena	1148	100	NE	150	43	15	present
11	Poggio Bastione 1 (Monte Nero-Poggio Bastione), Verghereto, Forlì-Cesena	1150	>100	N	120	51	11	present
12	Ripa Luna 1 (Alpe della Luna), Sansepolcro, Arezzo	1125	85	N	100	107	47	present
13	Ripa Luna 2 (Alpe della Luna), Badia Tedalda, Arezzo	1145	50	NE	50	39	18	present
14	Ripa Luna 3 (Alpe della Luna), Badia Tedalda, Arezzo	1150	85	NNW	450	151	84	present
15	Poggio Bastione 2 (Monte Nero-Poggio Bastione), Pieve S. Stefano, Arezzo	1125	90	NE	500	117	12	present
16	Ripa Bianca (Alpe della Luna), Badia Tedalda, Arezzo	1270	40	N	1800	138	70	present

**Table 2:** The multimodelling on GLM on the factor affecting mature and juvenile individuals abundance and density, and on *Cirsium alpis-lunae* cover at the subplot level. Averaged coefficients are provided only for factors included in the set of best models (see supplementary materials for full model selection tables). A (^+^) marks the log-transformed variables in the following analyses, while a £ marks those transformed with an *arcsin* transformation. Significance codes: *p* < 0.001 = ***; *p* < 0.01 = **; *p* < 0.05 = *.

Response	Variable	Factor Relative Importance *w_+(j)_*	Averaged Coefficient	Adjusted SE	z Value	Pr (>|z|)	
Abundance of juvenile individuals *	(Intercept)	-	0.74	0.80	0.92	0.358	
	Estimated area ^+^	1	0.49	0.09	5.66	<0.001	***
	Eastness	0.14	−0.06	0.21	0.31	0.758	
	Northness	0.11	0.05	0.21	0.23	0.818	
Abundance of mature individuals *	(Intercept)	-	0.29	0.95	0.30	0.763	
	Estimated area ^+^	1	0.49	0.16	3.03	0.002	**
	Eastness	0.28	−0.21	0.42	0.50	0.618	
Density of juvenile individuals	(Intercept)	-	0.24	0.07	3.52	<0.001	***
	Eastness	0.15	−0.02	0.07	0.28	0.783	
	Northness	0.11	0.01	0.20	0.20	0.844	
Density of mature individuals	(Intercept)	-	0.14	0.09	1.64	0.101	
	Northness	0.13	0.01	0.05	0.23	0.822	
Cover Cirsium ^£^	(Intercept)	-	0.76	0.21	3.52	<0.001	***
	Canopy cover ^£^	1	−0.41	0.08	5.03	<0.001	***
	Ground cover ^£^	0.19	0.06	0.16	0.38	0.706	
	Shrubs cover ^£^	0.1	−0.02	0.08	0.26	0.797	

## Data Availability

Data are contained within the article or supplementary material.

## Supplementary Materials

The following supporting information can be downloaded at: https://www.mdpi.com/article/10.3390/plants11050653/s1, Table S1: Model selection table for the multimodel analyses studying the effect of the site characteristics on the abundance of juvenile individuals of *C. alpis-lunae*. Only models with a ΔAICc < 4 were used to calculate the averaged coefficient estimates. Table S2: Model selection table for the multimodel analyses studying the effect of the site characteristics on the abundance of mature individuals of *C. alpis-lunae*. Only models with a ΔAICc < 4 were used to calculate the averaged coefficient estimates. Table S3: Model selection table for the multimodel analyses studying the effect of the site characteristics on the density of juvenile individuals of *C. alpis-lunae*. Only models with a ΔAICc < 4 were used to calculate the averaged coefficient estimates. Table S4: Model selection table for the multimodel analyses studying the effect of the site characteristics on the density of juvenile individuals of *C. alpis-lunae*. Only models with a ΔAICc < 4 were used to calculate the averaged coefficient estimates. Table S5: Model selection table for the multimodel analyses studying the effect of the cover of the different vegetation layers (i.e., ground, shrub, and canopy cover) on the cover of *C. alpis-lunae*. Only models with a ΔAICc < 4 were used to calculate the averaged coefficient estimates.
